# Socioeconomic Inequality in Childhood Obesity 

**Published:** 2017-08-15

**Authors:** Ghobad Moradi, Farideh Mostafavi, Namamali Azadi, Nader Esmaeilnasab, Ebrahim Ghaderi

**Affiliations:** ^1^ Social Determinants of Health Research Center, Kurdistan University of Medical Sciences, Sanandaj, Iran; ^2^ Student Research Committee, Kurdistan University of Medical Sciences, Sanandaj, Iran; ^3^ Social Determinants of Health Research Center, Iran University of Medical Sciences, Tehran, Iran

**Keywords:** Obesity, Children, Socioeconomic Status, Inequality

## Abstract

**Background:** The aim of this study was to assess the socioeconomic inequalities in obesity and overweight in children aged 10 to 12 yr old.

**Study design:** A cross-sectional study.

**Methods:** This study was conducted on 2506 children aged 10 to 12 yr old in the city of Sanandaj, western Iran in 2015. Body mass index (BMI) was calculated. Considering household situation and assets, socioeconomic status (SES) of the subjects was determined using Principal Component Analysis (PCA). Concentration Index was used to measure inequality and Oaxaca decomposition was used to determine the share of different determinants of inequality.

**Results:** The prevalence of overweight was 24.1% (95% CI: 22.4, 25.7). 11.5% (95% CI: 10.0, 12.0) were obese. The concentration index for overweight and obesity, respectively, was 0.10 (95% CI: 0.05, 0.15), and 0.07 (95% CI: 0.00, 0.14) which indicated inequality and a higher prevalence of obesity and overweight in higher SES. The results of Oaxaca decomposition suggested that socioeconomic factors accounted for 75.8% of existing inequalities. Residential area and mother education were the most important causes of inequality.

**Conclusions:** To reduce inequalities in childhood obesity, mother education must be promoted and special attention must be paid to residential areas and children gender.

## Introduction

Non-communicable diseases are the leading cause of death in most developed and developing countries. About 80% of deaths from non-communicable diseases occur in less-developed countries. The increasing prevalence of obesity and its related disorders in recent years has become one of the major health problems so that WHO has declared obesity as a growing epidemic^1^.

Obesity in children is rapidly increasing and alarming. Globally, of all the children living around the world, 20% are obese and 60% are overweight^2^. Eastern Mediterranean region has observed the highest prevalence of overweight and obesity worldwide and 10% of school-aged children are obese; the trend is still rising in this area^3^. In recent years, obesity and overweight among Iranian children have become a serious problem^4^. Given the high prevalence of obesity and overweight among school-aged children (17%), Iran has become one of the countries with the high prevalence. In Tehran, Iran 10% of adolescents had metabolic syndrome, which was mainly attributed to the high prevalence of obesity and overweight among this age group^5^.

Despite the increasing prevalence of obesity, due to its multidimensional nature, the interventions designed for the prevention of this problem had very limited achievements^6^. The most important way to reduce and control obesity is to identify factors associated with obesity. The experience of developed countries shows that focusing on individual behaviors is not a suitable approach to control the prevalence of obesity^7^. Socioeconomic status (SES) plays an important role in the development of diseases such as obesity. Socioeconomic factors such as poverty, education and occupation can affect people’s health by 50%, while the shares of the health system, genetic factors, and the environment is about 25%, 15%, and 10%, respectively^8^. The SES is one of the important indicators of health which can influence people’s attitude, behavior, and type of exposure to various risk factors^9, 10^.

Inequality in SES has led to differences in the prevalence of obesity and its associated complications in different countries. To decrease inequalities, first it is necessary to determine the link between social determinants of health and different health problems^11^. In high-income countries, obesity is more prevalent among the poor people and women while in poor countries it is more prevalent among high-income individuals. In less developed countries the burden of obesity has shifted toward people with lower SES^12^. Although recently there more attention has been paid to the prevalence of obesity among children, but to the best of our knowledge no study has been conducted in Iran to evaluate inequality in risk factors of non-communicable diseases in children. Studies that have addressed some aspects of SES in childhood obesity suggested an inverse association between obesity and their parents' level of education^13^.

This study aimed to determine the inequality in children obesity. We used Oaxaca decomposition to evaluate the share of different determinants of inequality in children obesity.

## Methods

This study was a cross-sectional study, conducted in 2015 in Sanandaj, the capital city of Kurdistan Province, the west of Iran. Based on the previous studies, social inequality of students is at least 20% (*P*=0.20). By accepting a 5% error rate, Z_1-α/2 is_   is 1.96. To determine the value of d, considering interval 0.5p ≤ d ≤ 0.2p, we assume d = 0.02 (10% p). We consider the design effect to be 1.5, which would result in a final sample size of 2,550 male and female students studying in the fifth and sixth grades of primary school (aged 10-12) yr old.

The subjects were chosen from the schools located in Sanandaj City. The sampling framework included two regions of Sanandaj Education and Training Office. The first region included 84 schools and the second included 42 schools. To select the samples, multistage sampling method was used. Accordingly, the two regions were selected as the two main strata, and the schools in each stratum were selected as clusters. The sample size allocated to each stratum was in proportion to the size of that stratum; accordingly, 1600 people from the first region and 900 people from the second region were enrolled into the study. In view of that, 39 schools (clusters) from the first region and 24 schools from the second region were selected. Moreover, within each school, again, the educational grades were selected as the main strata and the classes at each grade were considered as clusters of that stratum. The number of classes at each cluster and the number of students at each cluster were selected in proportion to their sizes. Finally, of the samples allocated to each stratum, the required number of the subjects was selected via random convenience sampling method.

Six interviewers conducted the survey. They were divided into two groups with three members in each group. In addition, a supervisor was assigned to monitor the interviews and examination. The interviewers and supervisors were trained prior to the initiation of the study. To collect the required data, students were interviewed and their parents completed questionnaires. First, the students were examined at school and were interviewed to complete a questionnaire. Then, parents with the cooperation of students completed the questionnaires at home or via phone calls.

This study was conducted according to the guidelines laid down in the Declaration of Helsinki and all procedures involving human subjects were approved by the Kurdistan University of Medical Sciences Ethics Committee (with 94/25 the committee’s reference number). Informed consent was obtained from all participants and their parents.

To measure the participants’ heights, they were set in a standing position, without shoes, with their feet flat together, with their shoulders level, and with their backs against a wall with a measuring tape on it. The heights were measured by touching the ruler on the top of the head. The participants’ weights were measured using a digital scale, with minimum clothing, with a precision of 100 grams. We also calculated the individual body mass index or BMI (weight in kg divided by the square of the height in meters). Following the recommendations by the WHO, to define overweight and obesity we used an age- and gender-specific BMI criteria used globally as a standard measure of obesity for children and adolescents aged 2-19 yr old. Accordingly, overweight was defined as a percentile from 85-95 and obesity was defined as a percentile over 95^3, 14^.

To determine the SES, we used a method proposed by O'Donnell. Accordingly, we used a questionnaire which included a number of questions about household assets including separate bathroom, separate kitchen, vacuum cleaners, computers, separate refrigerators, washing machine, color TV, LCD TV, mobile phone, dishwasher, microwave oven, internet access, personal car, landline telephone, personal home, number of rooms, heating appliances, oven, microwave, and furniture. Using principal composition analysis (PCA) method, asset index was calculated for each of the subjects, which ranks people from the poorest to the richest. . Based on this index and ranking weight, the studied population was divided into five quintiles, including very poor, poor, moderate, rich, and very rich. 51.19% of variation was explained by the component. Of these, the two groups of very poor and very rich were selected as the limits and socioeconomic inequalities were compared between these two groups^15, 16^.

The concentration curve and concentration index were used to measure inequality. If all the people in different socioeconomic classes have the same health status, the concentration index will become zero and the concentration curve will be tangent to the line of equality. When the desired variable is at the lowest SES level, CI will be negative and above the line of equality; however, when it is at the highest SES level, CI will be positive and below the line of equality^14^.

After measuring inequality using Oaxaca decomposition, we determined the share of each of different socioeconomic determinants and calculated the size of their effect on inequality. Oaxaca decomposition is used to determine the level of changes in Y caused by variations in the X inequality variable. The Oaxaca decomposition formula is as follows:

y^nonpoor^–y ^poor^=Δxβ^poor^+ Δβx^poor^+ΔxΔβ=E+C+CE

Oaxaca deconstructed the mean differences in outcome variable into two components; E component (explained) represents the differences in mean Xs or determinants, while C component (coefficients) represents differences in the mean coefficients, which include unexplained variables. Overall, CE represents the interaction between the differences in Xs and coefficients ^17^. To calculate the share of each of the components in the total difference, Oaxaca Decomposition formula was used as follows:

y^ nonpoor^–y ^poor^= (β_0_^nonpoor^-β_0_^poor^)+ (β_1_^nonpoor^x_1_^nonpoor^- β1^poor^x_1_^poor^) +(β_2_^nonpoor^x_2_^nonpoor^- β_2_^poor^x_2_^poor^) = G_0_+G_1_+G_2_

Where y is the gap or the mean change in the outcome variables i.e. the obesity and overweight. It can be thought of as being due in part to: G_0_ is the difference in the intercept; G_1_ is the difference in x_1_, β_1,_ for example, differences in economic attainment (x1) and the effects of economic attainment (β 1); and G_2_ is the difference in the x_2_, β_2_, for example, differences in the residential area attainment (x_2_) and the effects of the residential area attainment (β_2_).

To apply decomposition, first, using ordinary logistic regression we evaluated the relationship between outcome variables of obesity and overweight and different determinants such as age, gender, parental level of education, parental age, the residential area and economic groups. The variables with a significant relationship were entered into Oaxaca model. For all the models, the significance level was set at 0.05. Stata 13 software was used for the analysis of data ^14^.

## Results

Of 2506 participants enrolled into the study, 1284 were fifth grade students and 1224 were sixth grade students. Of all, 374 students were 10 yr old, 1044 students were 11 yr old, and 1088 students were 12 yr old. Of all, 24.1% (95% CI: 22.4, 25.7) were overweight and obese, 11.5% (95% CI: 10.0, 12.0) were obese, and 14.1% (95% CI: 12.0, 15.0) were lean. The prevalence of overweight and obesity was 25.4% (95% CI: 22.6, 28.2) in males and 23.3% (95% CI: 21.2, 25.4) in females. Moreover, 13.0% of boys (95% CI: 10.0, 15.0) and 10.0% of girls (95% CI: 9.0, 12.0) were obese.

The number of people in each quintile was as follows: 1120 people (44.0%) in the first quintile, 465 people (18.5%) in the second quintile, 356 people (14.2%) in the third quintile, 315 people (12.5%) in the fourth quintile, and 250 people (10.0%) in the fifth quintile. Table 1 shows the prevalence of overweight and obesity in terms of each of the independent variables including age, gender, parental level of education, parental age, and economic group. Ordinary logistic regression was used to obtain the odds ratios and to compare the outcome variable with different variables. The prevalence of overweight and obesity was higher in people living in areas with high socioeconomic level, based on the findings shown in Table 1, there was a direct relationship between residential area and overweight 1.68 (95% CI: 1.12, 2.35) and obesity 2.36 (95% CI: 1.37, 4.08). The prevalence of obesity was higher in boys than in girls as 0.76 (95% CI: 0.59, 0.98). Mother’s level of education was inversely associated with the prevalence of obesity as 0.59 (95% CI: 0.35, 0.99). The prevalence of obesity in people with high SES was higher than in those with low SES which was not significant. In addition, obesity had no significant association with parental age and father's level of education.

**Table 1:** Overweight and obesity according to different independent variable, Kurdistan, Iran, 2015

**Variables**	**Normal weight**	**Overweight**	**Obese**	**Overweight versus normal weight**		**Obese versus normal weight**	
				**Crude OR** **(95% CI)**	**Adjusted OR** **(95% CI)** ^a^	**Crude OR** **(95% CI)**	**Adjusted OR** **(95% CI)** ^a^
Sex							
Male	553	236	121	1.00	1.00	1.00	1.00
female	995	368	169	0.89 (0.74, 1.07)	0.85 (0.70, 1.04)	0.80 (0.62, 1.02)	0.77 (0.59, 0.99)
Mother Education							
Uneducated	295	91	56	1.00	1.00	1.00	1.00
Elementary	401	145	66	1.22 (0.91, 1.65)	1.11 (0.81, 1.53)	0.85 (0.58, 1.24)	0.82 (0.55, 1.23)
Guidance	241	116	51	1.68 (1.23, 2.30)	1.29 (0.91, 1.82)	1.07 (0.71, 1.60)	0.84 (0.54, 1.32)
High school	344	133	64	1.34 (0.99, 1.81)	0.95 (0.66, 1.35)	0.98 (0.67, 1.44)	0.72 (0.46, 1.14)
Academic	266	118	53	1.54 (1.13, 2.11)	0.99 (0.67, 1.48)	1.02 (0.68, 1.52)	0.62 (0.37, 0.99)
Father Education							
Uneducated	148	36	22	1.00	1.00	1.00	1.00
Elementary	322	94	46	1.16 (0.76, 1.78)	1.08 (0.69, 1.68)	0.90 (0.52, 1.53)	0.93 (0.53, 1.63)
Guidance	247	112	51	1.83 (1.21, 2.79)	1.58 (1.01, 2.47)	1.23 (0.72, 2.09)	1.23 (0.69, 2.18)
High school	349	146	67	1.76 (1.17, 2.63)	1.45 (0.92, 2.28)	1.19 (0.72, 1.99)	1.14 (0.64, 2.05)
Academic	477	214	103	1.86 (1.26, 2.75)	1.42 (0.89, 2.27)	1.32 (0.81, 2.15)	1.20 (0.66, 2.17)
Socioeconomic statue						
Richest SES	713	71	33	1.00	1.00	1.00	1.00
4th SES	287	82	45	1.20 (0.92, 1.55)	1.07 (0.82, 1.41)	1.19 (0.84, 1.68)	1.09 (0.76, 1.56)
Middle SES	203	105	47	1.58 (1.20, 2.07)	1.32 (1.00, 1.77)	1.38 (0.96, 1.98)	1.22 (0.82, 1.81)
2ndh SES	198	112	54	1.33 (0.99, 1.78)	1.09 (0.80, 1.50)	1.51 (1.04, 2.19)	1.29 (0.85, 1.94)
Poorest SES	147	134	111	1.55 (1.10, 2.04)	1.20 (0.85, 1.69)	1.38 (0.91, 2.09)	1.16 (0.73, 1.83)
Mother Age*							
<29	110	40	16	1.00	-	1.00	-
29-39	961	392	198	1.11 (0.76, 1.63)	-	1.42 (0.83, 2.44)	-
>40	476	171	76	0.98 (0.66, 1.46)	-	1.11 (0.63, 1.95)	-
Father Age							
<29	604	240	120	1.00	-	1.00	-
29-39	782	314	148	0.98 (0.81, 1.19)	-	0.92 (0.71, 1.19)	-
>40	156	48	21	0.74 (0.52, 1.05)	-	0.66 (0.41, 1.08)	-
Residential Area							
1	217	66	28	1.00	1.00	1.00	1.00
2	389	123	58	1.11 (0.80, 1.56)	1.07 (0.76, 1.50)	1.24 (0.77, 1.98)	1.15 (0.71, 1.86)
3	537	220	104	1.45 (1.06, 1.97)	1.29 (0.93, 1.78)	1.55 (1.00, 2.41)	1.43 (0.91, 2.27)
4	238	106	52	1.73 (1.22, 2.46)	1.50 (1.01, 2.21)	1.89 (1.16, 3.07)	1.82 (1.07, 3.11)
5	167	89	48	1.92 (1.33, 2.78)	1.64 (1.10, 2.46)	2.30 (1.40, 3.78)	2.25 (1.30, 3.87)
Age							
10	210	104	56	1.00	1.00	1.00	1.00
11	648	246	115	0.80 (0.61, 1.04)	0.81 (0.61, 1.06)	0.70 (0.49, 0.99)	0.70 (0.49, 0.99)
12	690	254	119	0.79 (0.60, 1.03)	0.81 (0.62, 1.06)	0.69 (0.49, 0.98)	0.71 (0.50, 1.01)

^a^ OR (crude) >0.2 didn’t enter in adjust model

**Figure 1:**
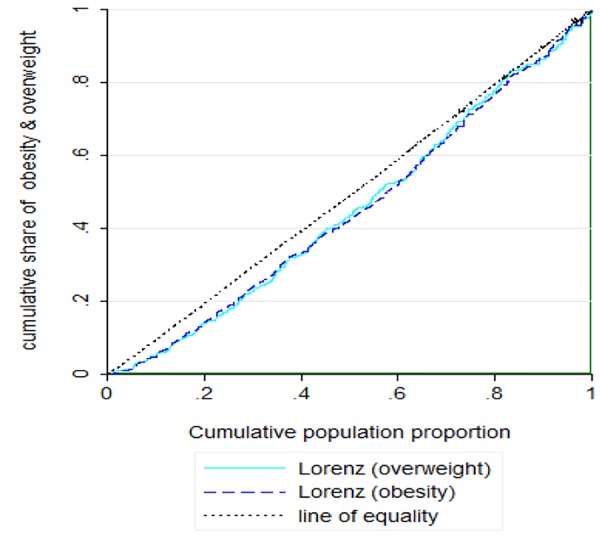
Concentration curve for obesity and overweight, Kurdistan, Iran, 2015

Table 2 presents the concentration index values for overweight and obesity. The concentration index for overweight and obesity was positive, which indicated inequality and a higher prevalence of obesity and overweight in higher socioeconomic groups. The inequality was also reflected in the concentration curve (Figure 1); the curve was placed under the line of equality. It indicates the prevalence of overweight and obesity in individuals with high socioeconomic status.

**Table 2:** Concentration index of obesity and overweight, Kurdistan, Iran, 2015

**Concentration Index**	**Coef.**	**Std. Err.**	**T**	**p>|t|**	**(95% CI)**	
Obesity	0.077	0.036	2.014	0.032	0.006	0.140
Overweight	0.100	0.026	3.860	0.001	0.050	0.150

The curve was placed under the line of equality. It indicates the prevalence of overweight and obesity in individuals with high socioeconomic status.

**Figure 2:**
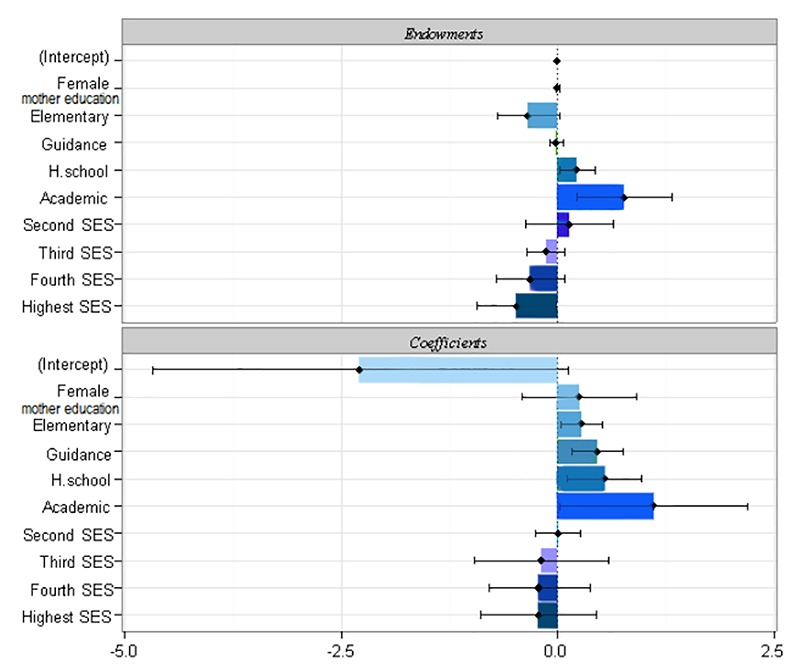
Contributions of differences in BMI and in coefficients to poorest, richest Oaxaca Decomposition, Kurdistan, Iran, 2015 (SES: socioeconomic status)
Obesity was more prevalent in the richest groups and low mother’s level of education

After determining the inequality, Oaxaca Decomposition was used to analyze the share of each of the determinants associated with obesity in creating inequality between rich and poor groups. As shown in Table 3, mean BMI was 17.6 (95% CI: 17.4, 17.8) for the poorest socioeconomic group and 18.4 (95% CI: 17.9, 18.8) for the richest group with better socioeconomic status. The gap between the two groups was 75.8%. Moreover, 55.8 or 73.7% of the total differences in BMI was due to explained component i.e. the difference in sex, mother’s level of education, and residential area; the largest share in the inequality was attributed to residential area (74.5%) and mother's level of education (23.6%). In the group with low SES, level of education had a protective role against the prevalence of obesity. Obesity was more prevalent in the richest groups where mother's level of education was low. The rest of the difference between the two socioeconomic groups i.e. 26.66% was attributed to unexplained component; it was attributed to differences in the coefficients or factors that had not been included in the study (Figure 2).

**Table 3:** decomposition of the difference in BMI between the richest and poorest groups, Kurdistan, Iran, 2015

**BMI**	**Prediction%**	**95% CI**	***P *** **value**
Mean BMI in the poorest group	17.64	17.44, 17.83	0.001
Mean BMI in the richest group	18.40	17.95, 18.84	0.001
Difference (Total Gap)	-0.76	-1.24, -0.27	0.002
**Due to endowment(explained)**		
Mother education	-0.13	-0.34, 0.07	0.200
Sex	0.00	-0.29, 0.11	0.400
Residential area	-0.41	-0.63, -0.19	0.001
Sub-total Gap (explained part)	-0.55	-0.81, -0.30	0.001
**Due to endowment (unexplained)**		
Mother education	0.98	-0.31, 2.29	0.130
Sex	-0.02	-0.69, 0.64	0.940
Residential area	-0.59	-2.28, 1.09	0.480
Sub-total Gap (unexplained part)	-0.20	-0.73, 0.33	0.460

## Discussion

This study is the first that used the Oaxaca decomposition to investigate inequalities in obesity in children. According to our results, the prevalence of obesity and overweight in children aged 10 to 12 yr were 11.5% and 24.1%, respectively. The results of Oaxaca analysis showed 75.8% of the gap between the richest and poorest groups was due to socioeconomic determinants or the explained factors; among these factors, the largest share in the inequality was attributed to residential area (74.5%) and mother's level of education (23.6%). The rest of the difference between the two socioeconomic groups i.e. 26.6% was attributed to unexplained component; it was attributed to differences in the coefficients or factors that had not been included in the study.

The results obtained for the concentration index suggested a positive significant inequality in obesity and overweight, which indicated a higher prevalence of obesity and overweight in higher socioeconomic groups. Therefore, overweight and obesity are more common in higher socioeconomic groups. It can be the result from excess calorie intakes, food preparation, eating practices, physical inactivity (i.e. screen time behavior, watching TV, playing electronic games) and cultural pattern favoring a larger body size in these groups. This pattern is consistent with the pattern of inequality in low-income countries. The results of our study is consistent with the results of Moradi^18^ OR=1.23 (95% CI: 0.73, 1.90), and Mocanu’s study in Romania^19^ OR=1.46 (95% CI: 1.10, 1.93). The Results of these studies, similar to our study, suggest a direct relationship between SES and obesity. Moreover, the results of studies which assessed the relationship between obesity and SES in Russia OR=1.20 (95% CI: 1.00, 1.40), and China OR=1.50 (95% CI: 1.00, 2.10) are also similar to results of our study^20^. However, the results of our study are inconsistent with the results of other studies ^21, 22^. In these two studies, unlike the results of our study, obesity was more prevalent among poor people.

As the results of the Oaxaca decomposition showed, residential area had the largest share in creating the gap between rich and poor groups. The prevalence of overweight and obesity was higher in areas with higher socioeconomic status. It results from different in physical inactivity pattern, available local area i.e. access to restaurants, types of food store by neighborhood characteristics and economic capacity to purchase these foods by children. Our findings are in line with the results of another study^23^, which reported a direct relationship between residential area and obesity. Nevertheless, our findings are not consistent with the results of studies by Sundblom et al. OR=1.91 (95% CI: 1.19, 2.55)^24^ and Jin Won Noh et al. OR=0.8 (95% CI: 0.7, 0.9) ^25^ which reported high prevalence of obesity in areas with lower SES.

Our results showed that, after the residential area, mother’s level of education was the second most important determinant of inequality. Educated mothers are affected by social and health standards. The prevalence of obesity and overweight was lower in children whose mothers had higher levels of education. Based on the findings of this study, in the group with low SES, mothers' level of education was a factor protecting children against obesity. In high SES groups, the prevalence of obesity was higher among children whose mother had a low level of education. Our finding is consistent with the result of Motlagh et al.’s study ^26^ which reported an association between mother’s level of education and obesity (*P*<0.05). However, our findings are not consistent with another study^25^ that reported a direct relationship between obesity and mother’s level of education.

The prevalence of overweight was 25.4% in males and 23.34% in females. Moreover, 13% of boys (95% CI: 10%, 15%) and 10% of girls (95% CI: 9%, 12%) were obese. In view of that, the prevalence of obesity was higher in boys than girls were; this finding is consistent with the results of previous studies^27, 28^.

Socioeconomic pattern of overweight and obesity and the prevalence of chronic diseases will lead to an increased level of health inequality in the future. Despite the stable prevalence of obesity in children and adolescents in Australia, Japan, France, UK, and the USA, the trend of obesity and overweight is not stable among all socioeconomic groups^29^.

The findings of different studies indicate that the obesity epidemic is manageable. The results of studies in America show that after years of steady increase in the prevalence of obesity, its trend has finally reached a constant level^14, 15^. During the years 1999 to 2010, the consumption of calories was decreased by 7% among boys aged 2-19 years old while it decreased by 4% among girls. Adolescents have higher levels of physical activity and consume more fruits and vegetables^19, 30^.

Due to developments in communities, in the future it is expected to observe higher prevalence of obesity among people with lower socioeconomic status. The current pattern of childhood obesity in Iran is largely similar to the patterns observed in developing countries. Compared with the other individual factors, the residence area plays an important role in inequality. Environmental factors associated with the location are among the factors that influence people’s behavior. Socioeconomic conditions of every location have an impact on several factors such as the type and concentration of high calorie and fast food supply centers; they can also affect children's dietary behaviors. Hence, one of the recommendations for the prevention of childhood obesity is to pay special attention to the residential areas and people’s location and design related interventions to reduce high-risk behaviors.

This study had some limitations. First, the study was conducted in Sanandaj City, which is a sample of the country, whoever it cannot be representative of all different regions of Iran, and the results cannot be completely generalized to Iran. Second, the data about the assets were collected via using a self-report questionnaire; as a result, the collected data might have some bias.

## Conclusions

The prevalence of obesity in children is directly associated with the socioeconomic status. There is little inequality in the distribution of obesity in the community, which represents higher prevalence of obesity in higher socioeconomic groups. To reduce inequalities in childhood obesity special attention must be paid to mother’s level of education, and special intervention must be designed for the residential area and gender of children.

## Acknowledgment

This paper was extracted from an MS thesis.This study was approved and supported by Kurdistan University of Medical Sciences.

## Conflict of interest statement

The authors declare that they have no conflict of interest‏.

## Funding

This study was supported by Kurdistan University of Medical Sciences‏.

## Highlights

- The concentration index for overweight and obesity indicated inequality and a higher prevalence of obesity and overweight in higher socioeconomic groups.
- The gap between the two groups was 75.87%.
- The largest share in the inequality was attributed to residential area and mother's level of education.
- In the group with low SES, level of education had a protective role against the prevalence of obesity.
- Obesity was more prevalent in the richest groups where mother's level of education was low.
